# Simulated Oxygen Supply Efficiency Assessment to Represent Stored Red Blood Cells Quality

**DOI:** 10.3390/life16020205

**Published:** 2026-01-26

**Authors:** Zongtang Chu, Guoxing You, Weidan Li, Peilin Shu, Dong Qin, Lian Zhao, Hong Zhou, Ying Wang

**Affiliations:** Academy of Military Medical Sciences, Beijing 100850, China; m18613105106@163.com (Z.C.); bjguoxingyou@163.com (G.Y.); lwd_dan@163.com (W.L.); yk2748764926@163.com (P.S.); 18893191599@163.com (D.Q.); zhouhtt1966@163.com (H.Z.)

**Keywords:** P_50_, Hill coefficient, acid-base sensitivity index, theoretical oxygen release capacity, stored red blood cells, oxygen supply efficiency

## Abstract

Hemolysis rate is usually used as the acceptance criterion for stored red blood cells (RBCs) in clinical practice. However, there is a current lack of parameters for the characterization of hemoglobin quality. This study aimed to incorporate oxygen affinity, cooperativity, and the Bohr effect into a parameter system to monitor oxygen supply efficiency in stored RBCs, potentially serving as a basis for quality assessment. Han Chinese blood from plains, Tibetan blood from plateau, bovine hemoglobin (bHb), and a dextran–bovine hemoglobin conjugate (Dex20-bHb) were analyzed using the BLOODOX-2018. Oxygen affinity (P_50_) was determined by oxygen dissociation curves (ODCs) at pH = 7.4. Cooperativity was assessed through the Hill coefficient, calculated from the fitting range of the Hill equation. The Bohr effect was evaluated by the acid-base sensitivity index (SI) under simulated pH conditions of the lungs (pH = 7.6) and tissues (pH = 7.2) to calculate corresponding P_50_ values. Oxygen partial pressures (PO_2_) simulating lungs (PO_2_ = 100 mmHg for plains and 60 mmHg for plateau) and tissues (PO_2_ = 40 mmHg for plains and 30 mmHg for plateau) were used to calculate theoretical oxygen-release capacities in both environments. Multiple regression analysis explored relationships among parameters, constructing a system to assess changes in rat RBCs during storage. Optimized test methods determined P_50_, Hill coefficient, SI, and theoretical oxygen-release capacities for Han Chinese blood, Tibetan blood, bHb, and Dex20-bHb samples in various environments. We constructed a parameter system to characterize blood’s oxygen supply efficiency, revealing the significant influence of the Bohr effect. This influence varied with environmental changes in oxygen affinity. We validated the system using stored rat RBCs, finding consistent P_50_ trends with predictions, and initial increases in Hill coefficient and SI followed by decreases. Theoretical oxygen-release capacities varied significantly between plateau and plain environments. These results support using oxygen supply efficiency to assess RBC storage quality for developing transfusion strategies. P_50_, Hill coefficient, SI, and theoretical oxygen-release capacity in different environments can be incorporated into blood oxygen supply efficiency characterization systems to assess the quality changes in RBCs during storage.

## 1. Introduction

Transfusion is the primary modality for treating massive blood loss and severe anemia in clinical practice. It utilizes the oxygen-binding/release capacities of hemoglobin (Hb) in stored red blood cells (RBCs) to alleviate the insufficiency in oxygen supply caused by blood loss or anemia. The Hb level in patients is mainly used as the basis for transfusion and assessment of transfusion effectiveness in clinical practice [1,2]. However, RBCs undergo a series of changes during the storage period, including a reduction in deformability and accumulation of metabolites [3,4]. Hemolysis rate is presently the sole parameter used for characterizing stored RBCs, but researchers have become increasingly aware of the fact that changes also occur in the oxygen supply efficiency of blood during storage, which may influence the outcomes of transfusion [5,6]. In clinical practice, it has been found that differences exist in transfusion outcomes even when transfusion is performed with equivalent amounts of Hb [7]. Ineffective rate of red blood cell transfusion may be as high as 18.2%, with transfusion ineffective rate reaching 37% and 42.8%, respectively, in patients with hematological disorders and malignant tumors [8]. Besides known immune factors and drug effects, differences in RBCs’ oxygen supply efficiency may also be a key contributing factor to this phenomenon.

There is a current lack of research on the oxygen supply efficiency of stored RBCs. Existing studies have mostly assessed oxygen supply efficiency using the oxygen affinity of Hb under standard conditions (pH = 7.4, PCO_2_ = 40 mmHg). Oxygen affinity reflects the oxygen-binding/release capacities of Hb and is characterized by P_50_, i.e., the oxygen partial pressure corresponding to 50% oxygen saturation (SO_2_) on the oxygen dissociation curve (ODC) [9]. With the deepening of research, there has been increasing awareness among researchers regarding the inadequacy of oxygen affinity under standard conditions in representing the oxygen supply efficiency of blood. Previous research has revealed that during their evolutionary process, penguins exhibited increased blood oxygen affinity and oxygen-binding capacity, enabling enhanced oxygen extraction from the lungs. Although heightened oxygen-binding capacity generally reduces oxygen release from tissue capillaries, penguins display a pronounced Bohr effect that, conversely, facilitates increased oxygen delivery to tissue capillaries. This adaptation enhances hypoxic tolerance and extends the duration of underwater foraging trips [10]. This phenomenon underscores the necessity for refined parameter systems to evaluate the efficiency of oxygen supply.

Cooperativity and the Bohr effect are critical characteristics of Hb [11,12]. In humans and most mammals, Hb’s unique tetrameric structure enables each molecule to bind four oxygen molecules. Oxygen binding triggers conformational changes in Hb, enhancing the binding of subsequent oxygen molecules, a process that initially progresses slowly but accelerates subsequently. This phenomenon, known as cooperativity, produces the characteristic S-shaped ODC for Hb [13,14]. The Bohr effect involves the binding of H^+^ to Hb, promoting the formation of salt bridges and a conformational shift to the T-state, which decreases Hb’s oxygen affinity. Conversely, a decrease in acidity disrupts these bridges, transitioning Hb to the R-state and increasing its oxygen affinity. The Bohr effect enables oxygen loading in the lungs—where pH is higher—and unloading in tissue capillaries, where pH is lower. Thus, both cooperativity and the Bohr effect are vital for the efficient oxygen supply of blood and should be integral to any parameter system evaluation [15].

In the present study, whole blood from Han Chinese in plain regions (Han Chinese blood), whole blood from Tibetans in plateau (Tibetan blood), bovine Hb (bHb), and a dextran–bovine Hb conjugate (Dex20-bHb) were systematically analyzed for factors influencing Hb properties such as oxygen affinity, cooperativity, and the Bohr effect. A system of parameters for evaluating the oxygen supply efficiency of blood was established by selecting appropriate characterization parameters, refining testing methods, and identifying correlations among these parameters. Tests were then conducted on stored rat RBCs to explore variations in their oxygen supply efficiency. Our findings aim to provide a proof-of-concept analytical tool for assessing RBCs’ storage quality.

## 2. Materials and Methods

### 2.1. Instruments and Reagents

Thirty healthy male specific-pathogen-free Wistar rats weighing 220–260 g each were provided by Beijing Charles River Laboratories and maintained in the animal facility at the Academy of Military Medical Sciences. The protocol was approved by the Laboratory Animal Center of the Academy of Military Medical Sciences (IACUC-DWZX-2022–631, Beijing, China).

The following reagents and instruments were used in this study: bovine whole blood (Created Biotechnology, Beijing, China), sodium hydroxide (NaOH; Sinopharm, Shanghai, China), potassium chloride (KCl; Sinopharm, China), TES buffer (Thermo Fisher, Waltham, MA, USA), bovine serum albumin (BSA; Thermo Fisher, Waltham, MA, USA), Dimethylpolysiloxane (Thermo Fisher, Waltham, MA, USA), citrate-phosphate-dextrose-adenine-1 (CPDA-1) cell preservation buffer (Thermo Fisher, USA), leukoreduction filter (Bengbu Medical College, Bengbu, China), BLOODOX-2018 oxygen binding-dissociation analyzer ( Softron Biotechnology, Beijing, China), and ӒKTA pure chromatography system (GE Health, Marlborough, MA, USA).

### 2.2. Sample Preparation

#### 2.2.1. Bovine Hb (bHb)

Bovine whole blood was subjected to separation, cleaning, hemolysis, microfiltration, chromatography, and ultrafiltration to obtain purified bHb. After adjustment of the concentration to 11–13 g/dL, the bHb was aseptically aliquoted and stored at −80 °C [16].

#### 2.2.2. Dextran–Bovine Hb Conjugate (Dex20-bHb)

Dex20-bHb was prepared in accordance with previously reported methods [17,18,19]. Dextran (0.5 mM, 20 mL) was oxidized by 10 mM sodium periodate in 10 mM sodium acetate buffer (pH 5.8). The reaction was kept in dark conditions for 90 min at room temperature and stopped by the addition of excessive ethylene glycol, followed by extensive dialysis against PBS buffer (pH 7.4) at 4 °C. At the same time, 0.25 mM bHb (20 mL) was allowed to react with 1 mM 4-PDS in PBS buffer (pH 7.4) for 3 h at 4 °C. The resultant bHb (320 mg) was incubated with the oxidized dextran (320 mg) and sodium cyanoborohydride (160 mg) overnight at 4 °C. Afterwards, 4-fold molar excess of TCEP over bHb was added to release the thiopyridyl moiety from the disulfide moiety for 40 min at 4 °C. The unreacted dextran and bHb were removed from the conjugate with protection of Cys-93(β) (Dex-bHb) by a tangential flow filtration instrument with a 100 kDa cutoff membrane.

#### 2.2.3. Whole Blood from Han Chinese and Tibetans

Human whole-blood samples were withdrawn from the median cubital veins of volunteers who were six healthy Han Chinese and six healthy Tibetan men, 28–36 years old, who gave their informed consent. All personnel had signed an informed consent form prior to the experiment and were screened for blood donors (including routine blood tests, four infectious diseases, and alanine aminotransferase (ALT)), which all met the relevant criteria. Then, 5 mL of blood was mixed with citrate phosphate dextrose adenine (CPDA-1; Sigma Aldrich, St. Louis, MO, USA), and the final concentration of CPDA-1 was 14%. The collection of human blood has been approved by the Ethics Committee (approval no. L2023002).

### 2.3. Plotting of Oxygen Dissociation Curves and Measurement of Oxygen Affinity

Following the methodology used in our previous research [20], different types of samples were first quantified for hemoglobin concentration (ctHb). Samples equivalent to 3 mg of Hb or an equal volume of RBCs were added to 4 mL of oxygen binding-dissociation analyzer buffer including 130 mM NaCl, 30 mM TES, and 5 mM KCl, with respective pH values of 7.2, 7.4, and 7.6. Subsequently, 20 μL each of BSA solution and Dimethylpolysiloxane were added, and the ODCs of the samples during the deoxygenation process were plotted in real time at 37 °C using the BLOODOX-2018 oxygen binding-dissociation analyzer. P_50_ values were read off the curves, with P_50_ at pH = 7.4 used for the characterization of oxygen affinity, and P_50_ at pH = 7.2 and 7.6 used for the calculation of the Bohr effect.

### 2.4. Optimization of Characterization Method for Cooperativity

The Hill coefficient is typically employed for the characterization of cooperativity. Calculations were performed to obtain the Hill coefficient to fit the experimental data from the ODC at pH = 7.4 obtained in Section 2.3 to the original and log-transformed Hill equations (Equations (1) and (2) below). However, the S-shape of the ODC indicates that the trends of change in the initial and terminal sections differed from the middle section, potentially lowering the degree of fit if the entire curve range was included in the fitting process. To address this issue, SO_2_ ranges of 1–99%, 20–80%, and 40–60% were selected, and the corresponding Hill coefficients and goodness of fit were obtained. Based on these results, the optimal fitting range was further selected, and cooperativity was determined by calculating the Hill coefficients of the various samples within this range.

(1) $$y=\frac{kp^{n}}{1+kp^{n}}$$

(2) $$\log\frac{y}{1-y}=n\log p+k$$

In the above equations, y is the oxygen saturation (SO_2_), p is the oxygen partial pressure (PO_2_), the slope *n* is the Hill coefficient, and the intercept k is the dissociation constant.

### 2.5. Selection of Characterization Method and Calculation of the Bohr Effect

The strength of the Bohr effect is typically characterized by the acid-base sensitivity index (SI), which is calculated by Equation (3) [21,22]. Changes in P_50_ under different pH environments were determined for the calculation of SI, which was, in turn, used for the assessment of the Bohr effect. In the present study, the pH environments of tissue capillaries and the lungs were simulated with pH values of 7.2 and 7.6, respectively. P_50_ values at pH = 7.2, 7.4, and 7.6 were denoted as P_50 acidic_, P_50 neutral_, and P_50 base_, respectively, and substituted into Equation (3) below to determine the SI values, which were ultimately used to characterize the strength of the Bohr effect among the various samples.

(3) SI = (P_50 acid_ − P_50 base_)/P_50 neutral_

### 2.6. Establishment of the Calculation Method for Theoretical Oxygen-Release Capacity

Theoretical oxygen-release capacity is defined as the amount of oxygen bound to blood in the lungs that can be subsequently released at the tissue capillaries, i.e., the difference in SO_2_ between these two internal environments. During the development of the calculation method for theoretical oxygen-release capacity, the following environmental settings were adopted: (1) PO_2_ ranges were introduced for the simulated plain and plateau environments, with 100 mmHg and 60 mmHg, respectively, representing the PO_2_ in the lungs in plain and plateau environments, and 40 mmHg and 30 mmHg, respectively, representing the PO_2_ in the tissue cells in these environments. (2) Considering the influence of the Bohr effect, the previously established buffer systems with different pH values were used as follows: the buffer system with pH = 7.4 represented the standard environment within the body, i.e., the standard evaluation system commonly used; the system with pH = 7.2 represented the pH environment of tissue capillaries, while the system with pH = 7.6 represented the pH environment of the lungs. The difference in blood SO_2_ between the lungs and tissue capillaries under the corresponding conditions was deemed the theoretical oxygen-release capacity. Six samples (*n* = 6) were analyzed in parallel at five different time points.

Using this method, the theoretical oxygen-release capacities of the various samples in different environments were calculated, as shown in Equations (4) and (5):

(4) ΔSO_2_ = SO_2(100 mmHg, pH = 7.6)_ − SO_2(40 mmHg, pH = 7.2)_

(5) ΔSO_2_′ = SO_2(60 mmHg, pH = 7.6)_ − SO_2(30 mmHg, pH = 7.2)_

where ΔSO_2_ is the calculated theoretical oxygen-release capacity in the simulated plain environment, and ΔSO_2_′ is the calculated theoretical oxygen-release capacity in the simulated plateau environment.

### 2.7. Assessment of Oxygen Supply Efficiency of Rat RBCs During the Storage Process

Fresh rat whole blood was obtained, preserved in CPDA-1, subjected to leukoreduction by filtration, and centrifuged at 400× *g* for 15 min [23]. After centrifugation, the supernatant was discarded, and the RBCs were aliquoted and stored at 4 °C. The hemolysis rate was measured at 0 d, 1 d, 3 d, 7 d, and 14 d of storage to determine the quality of the stored RBCs. The free Hb concentration (FHb) of the RBC samples was measured using a microhemoglobin detector (DiAspect Tm, Barleben, Germany). The hematocrit (HCT) and Hb concentration of RBCs were measured using a blood gas analyzer (ABL90 FLEX, Radiometer, Brønshøj, Copenhagen, Denmark) and the hemolysis rate of RBCs was calculated according to Equation (6).

(6) Hemolysis Rate (%) = [Free hemoglobin × (1 − Hct)]/ctHb

Oxygen supply efficiency-related parameters were calculated at the corresponding time points in accordance with the methods described in Section 2.3, Section 2.4, Section 2.5 and Section 2.6 to obtain the patterns of change in P_50_, Hill coefficient, SI, and theoretical oxygen-release capacity during the RBCs storage process. Six samples (*n* = 6) were analyzed in parallel at five different time points.

### 2.8. Statistical Analysis

Statistical analysis was performed using SPSS 22.0. Measurement data were presented as mean ± standard deviation ( $\bar{x}$ ± s). Intergroup comparisons of P_50_, Hill coefficient, SI, and theoretical oxygen-release capacities under different conditions of the four groups of samples (Han Chinese blood, Tibetan blood, bHb, and Dex20-bHb) were performed by one-way analysis of variance (ANOVA). Intragroup comparisons of theoretical oxygen-release capacities under simulated plain and plateau environments were conducted using the *t*-test. Normality and homogeneity of variance tests were conducted on all data as a prerequisite for statistical analysis. The Shapiro–Wilk test was employed to evaluate the normality assumption. Levene’s test was conducted to evaluate the equality of variances. The correlations of P_50_, Hill coefficient, and SI with theoretical oxygen-release capacity were determined via regression analysis. Differences were considered statistically significant when *p* < 0.05 and highly significant when *p* < 0.01.

## 3. Results

### 3.1. Construction of Parameter System for Characterizing Oxygen Supply Efficiency

#### 3.1.1. Oxygen Dissociation Curves (pH = 7.4) and Oxygen Affinity

**Figure 1:**
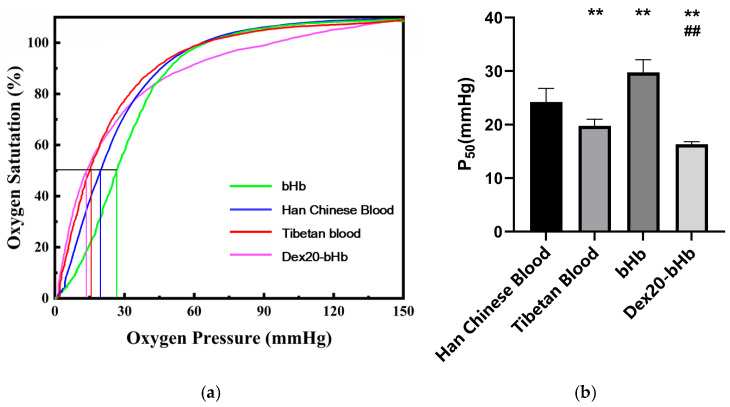
Oxygen dissociation curves (**a**) and P_50_ values of the four types of samples (**b**) (*n* = 6). **: *p* < 0.05 vs. Han Chinese blood; ##: *p* < 0.01 vs. bHb.

Figure 1a shows the S-shaped ODC (pH = 7.4) for the four types of samples. Compared with the ODC for Han Chinese blood, the curve for bHb was shifted to the right, while those for Tibetan blood and Dex20-bHb exhibited a leftward shift. As shown in Figure 1b, the P_50_ values for Han Chinese blood and Tibetan blood were (24.23 ± 2.56) mmHg and (19.77 ± 1.22) mmHg, respectively. Therefore, the oxygen affinity of Tibetan blood was higher than that of Han Chinese blood, with the difference being highly statistically significant (*p* < 0.01). The P_50_ values of bHb and Dex20-bHb were (29.75 ± 2.37) mmHg and (16.35 ± 0.47) mmHg, respectively, with the difference being highly statistically significant (*p* < 0.01). This result demonstrates that modification of bHb led to an increase in oxygen affinity.

#### 3.1.2. Optimization of a Measurement Method for the Hill Coefficient

**Figure 2:**
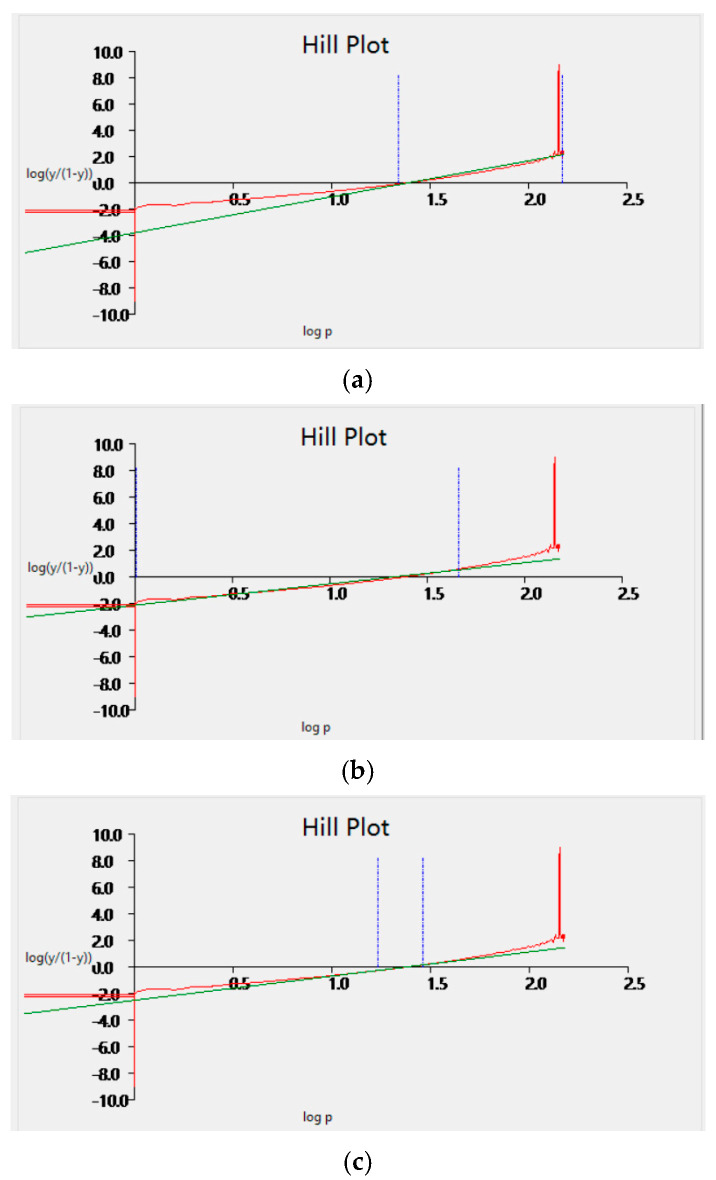
Fitting of Hill equation for different SO_2_ ranges using Han Chinese blood samples, SO_2_ = 1–99% (**a**), 40–60% (**b**) and 20–80% (**c**).

The optimal fitting range for the Hill coefficient was screened using the Han Chinese blood samples. Figure 2 shows that for the fitting ranges with SO_2_ = 1–99% (Figure 2a), 40–60% (Figure 2b) and 20–80% (Figure 2c) the Hill coefficients for Han Chinese blood were 1.91 ± 0.55, 2.43 ± 0.57, and 2.27 ± 0.21, respectively, and the corresponding goodness of fit values were 0.747 ± 0.126, 0.987 ± 0.012, and 0.995 ± 0.003. Therefore, the fitting range of 20–80% was used for subsequent measurements of the Hill coefficient.

**Figure 3:**
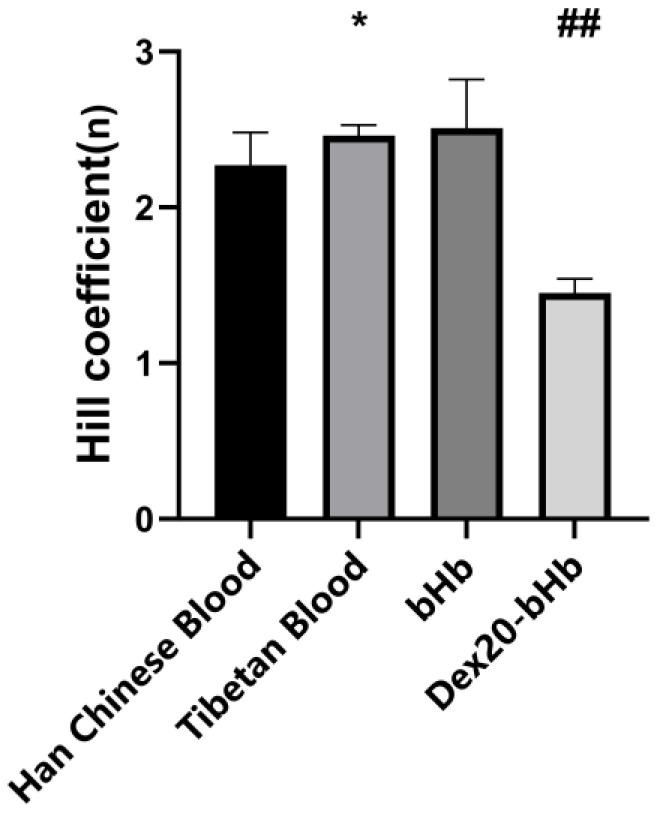
Hill coefficient values of the four types of samples (*n* = 6). *: *p* < 0.05 vs. Han Chinese blood; ##: *p* < 0.01 vs. bHb.

As shown in Figure 3, the Hill coefficients for Han Chinese blood and Tibetan blood were 2.27 ± 0.21 and 2.46 ± 0.07, respectively. Therefore, cooperativity was stronger in Tibetan blood, with the difference being statistically significant (*p* < 0.05). The Hill coefficients of bHb and Dex20-bHb were 2.51 ± 0.31 and 1.45 ± 0.09, respectively. This demonstrated that modification reduced the cooperativity of bHb, with the difference being highly statistically significant (*p* < 0.01).

#### 3.1.3. The Acid-Base Sensitivity Index (SI)

**Figure 4:**
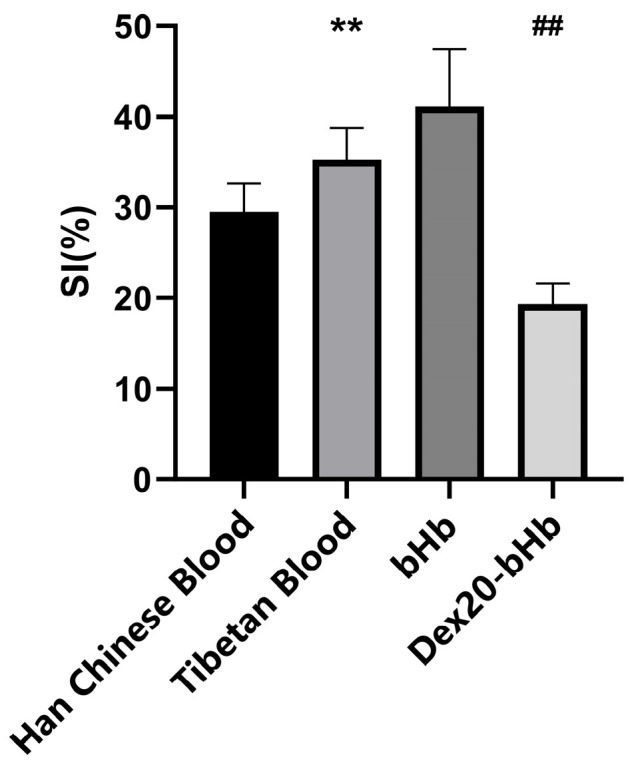
SI values for the four types of samples (*n* = 6). **: *p* < 0.01 vs. Han Chinese blood; ##: *p* < 0.01 vs. bHb.

As shown in Figure 4, SI values of Han Chinese blood and Tibetan blood calculated using Equation (3) were (29.51 ± 3.19)% and (35.28 ± 3.55)%, respectively. This indicated the presence of a stronger Bohr effect in Tibetan blood, with the difference being highly significant (*p* < 0.01). The corresponding SI values for bHb and Dex20-bHb were (41.15 ± 6.31)% and (19.35 ± 2.28)%, respectively, indicating that bHb modification weakened the Bohr effect to a highly significant extent (*p* < 0.01).

#### 3.1.4. Theoretical Oxygen-Release Capacity

**Figure 5:**
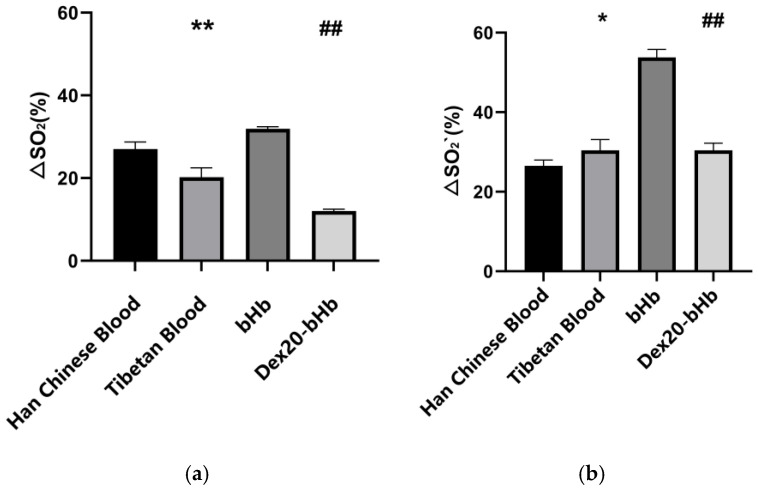
Calculated theoretical oxygen-release capacity values (*n* = 6) in the simulated plain environment (**a**) and plateau environment (**b**). *: *p* < 0.05 vs. Han Chinese blood; **: *p* < 0.01 vs. Han Chinese blood; ##: *p* < 0.01 vs. bHb.

Under the conditions of the simulated internal pH environments of the body (pH = 7.2, pH = 7.6), the theoretical oxygen-release capacities of Han Chinese blood and Tibetan blood in the simulated plain environment were (29.51 ± 1.82)% and (27.40 ± 3.32)%, respectively, which were not significantly different (*p* > 0.05). The theoretical oxygen-release capacities of bHb and Dex20-bHb in the simulated plain environment were (48.78 ± 1.74)% and (21.40 ± 1.81)%, respectively, with the difference being highly statistically significant (*p* < 0.01 Figure 5a).

Under the conditions of the simulated internal pH environments of the body (pH = 7.2, pH = 7.6), the theoretical oxygen-release capacities of Han Chinese blood and Tibetan blood in the simulated plateau environment were (26.51 ± 1.45)% and (30.42 ± 2.73)%, respectively. Therefore, the oxygen release capacity of Tibetan blood in the plateau environment was significantly higher than that of Han Chinese blood (*p* < 0.05). The theoretical oxygen-release capacities of bHb and Dex20-bHb in the simulated plateau environment were (53.78 ± 2.01)% and (30.42 ± 1.79)%, respectively, with the difference being highly statistically significant (*p* < 0.01) (Figure 5b).

#### 3.1.5. Analysis of Correlations Among Parameters

Oxygen affinity, cooperativity, the Bohr effect, and theoretical oxygen-release capacity were components of the parameter system for characterizing oxygen supply efficiency. Multiple regression analysis was performed to investigate the correlations among these parameters. Mathematical models were constructed by setting ΔSO_2_ and ΔSO_2_′, respectively, calculated using theoretical oxygen-release capacities in the plain and plateau environments, as the dependent variables, and P_50_, Hill coefficient, and SI as the independent variables. The ANOVA table and regression coefficients are provided in the Supplementary Materials.

It was found that both ΔSO_2_ and ΔSO_2_′ exhibited a linear relationship with P_50_, Hill coefficient, and SI. The mathematical models with the smallest residuals were as follows:

(7) *Y* = 5.501 + 0.588*X*_1_ + 10.839*X*_2_ + 0.032*X*_3_

(8) *Y′* = 4.493 + 0.146*X*_1_ + 2.152*X*_2_ + 0.511*X*_3_

*Y*: ΔSO_2_; Y′: ΔSO_2_′; *X*_1_: P_50_; *X*_2_: Hill coefficient; *X*_3_: SI

Results of statistical testing of Equation (7) revealed that *F* = 145.253 and *R*^2^ = 0.978, i.e., 97.8% of the changes in ΔSO_2_ could be explained by changes in P_50_, Hill coefficient, and SI. At the 5% significance level, the critical value of the *F* statistic was *F*_0.05_(12,3) = 3.490. Therefore, the value of the *F* statistic when the null hypothesis of the model held true (145.253) > *F*_0.05_(12,3), indicating that the linear relationship of the model was significantly established.

Results of statistical testing of Equation (8) revealed that *F* = 143.63 and *R*^2^ = 0.980, i.e., 98.0% of the changes in ΔSO_2_′ could be explained by changes in P_50_, Hill coefficient, and SI. At the 5% significance level, the critical value of the *F* statistic was *F*_0.05_(12,3) = 3.490. Therefore, the value of the *F* statistic when the null hypothesis of the model held true (143.63) > *F*_0.05_(12,3), indicating that the linear relationship of the model was significantly established.

The standardized regression coefficients show that cooperativity had a greater influence on the calculated theoretical oxygen-release capacity in Equation (7), i.e., the plain environment, while the Bohr effect had a greater influence on the calculated theoretical oxygen-release capacity in Equation (8), i.e., the plateau environment.

### 3.2. Assessment of Oxygen Supply Efficiency of Stored RBCs

#### 3.2.1. Changes in Hemolysis Rate of Stored RBCs

**Figure 6:**
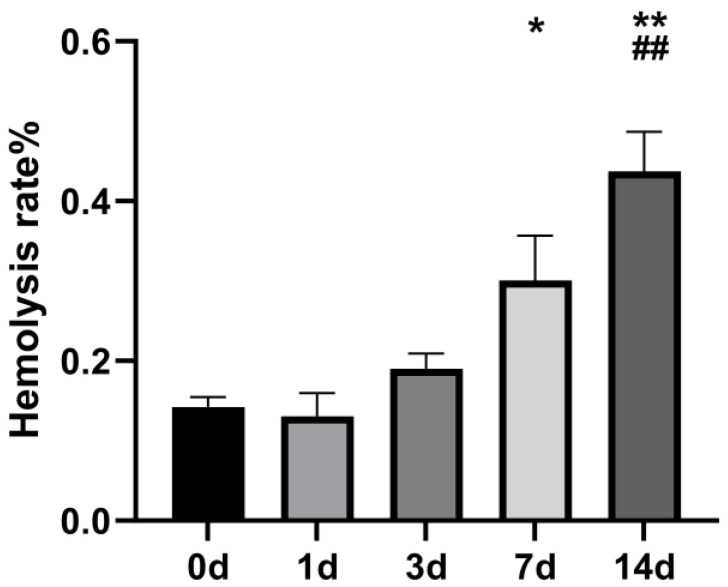
Changes in hemolysis rate of stored rat RBCs (*n* = 6). *: *p* < 0.05 vs. 0 d; **: *p* < 0.01 vs. 0 d; ##: *p* < 0.01 vs. 7 d.

As shown in Figure 6, the hemolysis rate of rat RBCs at 7 d was (0.30 ± 0.06)%, which was higher than that at 0 d (*p* < 0.05). At 14 d, the hemolysis rate was (0.44 ± 0.05)%, which was highly significantly elevated compared with 0 d and 7 d (*p* < 0.01) but still within the limit of ≤0.8% for stored RBCs stipulated by the Chinese standard GB 18469-2012: Quality Requirements for Whole Blood and Blood Components [24].

#### 3.2.2. Changes in Oxygen Dissociation Curve and Oxygen Affinity of Stored RBCs

**Figure 7:**
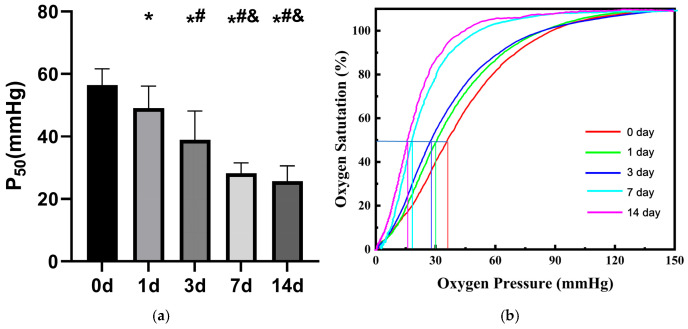
Changes in oxygen dissociation curve (**b**) and P_50_ of stored rat RBCs (**a**) (*n* = 6). *: *p* < 0.05 vs. 0 d; #: *p* < 0.05 vs. 1 d; &: *p* < 0.05 vs. 3 d.

As shown in Figure 7b, in a pH 7.4 environment, the ODC of RBCs progressively shifted leftward over time. Concurrently, the P_50_ values decreased and oxygen affinity increased as storage time extended. Specifically, the P_50_ values at 0 d, 1 d, 3 d, 7 d, and 14 d were (56.42 ± 5.24) mmHg, (49.11 ± 7.02) mmHg, (38.89 ± 9.29) mmHg, (28.21 ± 3.27) mmHg, and (25.69 ± 4.88) mmHg, respectively (Figure 7a). Relative to the initial P_50_ value at 0 d, there was a 12.96% decrease by 1 d (*p* < 0.05), 31.07% decrease by 3 d (*p* < 0.05), 50.01% decrease by 7 d (*p* < 0.05), and 54.47% decrease by 14 d (*p* < 0.05). However, the reduction in P_50_ between 7 d and 14 d did not reach statistical significance (*p* > 0.05).

#### 3.2.3. Changes in Cooperativity and the Bohr Effect of Stored RBCs

**Figure 8:**
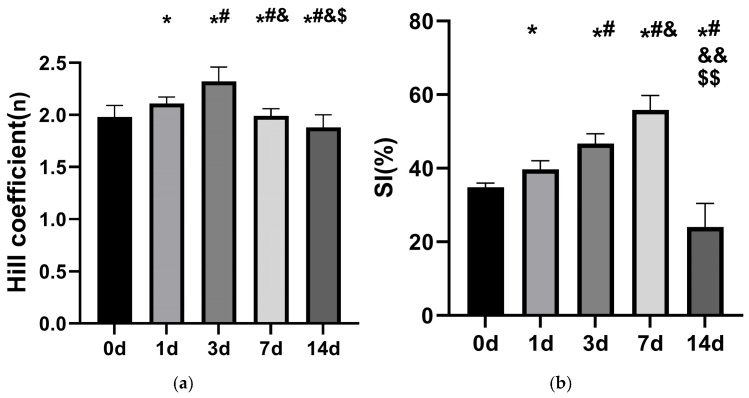
Changes in the cooperative effect (**a**) and Bohr effect (**b**) of stored rat RBCs (*n* = 6). *: *p* < 0.05 vs. 0d; #: *p* < 0.05 vs. 1 d; &: *p* < 0.05 vs. 3 d; &&: *p* < 0.01 vs. 3 d; $: *p* < 0.05 vs. 7 d; $$: *p* < 0.01 vs. 7 d.

In a pH 7.4 environment, the Hill coefficient values for stored RBCs at 0 d, 1 d, 3 d, 7 d, and 14 d were 1.98 ± 0.11, 2.11 ± 0.06, 2.32 ± 0.14, 1.99 ± 0.07, and 1.88 ± 0.12, respectively (Figure 8a). These data suggest a trend of increasing, then decreasing cooperativity over time. Notably, a significant rise occurred from 0 d to 3 d (*p* < 0.05), peaking at 3 d. Thereafter, values gradually declined through 14 d (*p* < 0.05), with the 7 d value falling below that of 0 d (*p* < 0.05). The SI values for stored RBCs at 0 d, 1 d, 3 d, 7 d, and 14 d were 34.82 ± 1.17%, 39.73 ± 2.28%, 46.73 ± 2.61%, 55.82 ± 3.95%, and 24.01 ± 6.42%, respectively (Figure 8b). This pattern reflects an initial increase followed by a subsequent decrease in the Bohr effect. A statistically significant rise was observed from 0 d to 3 d, followed by a significant decline from 3 d to 14 d (*p* < 0.05).

#### 3.2.4. Changes in Theoretical Oxygen-Release Capacity of Stored RBCs

**Figure 9:**
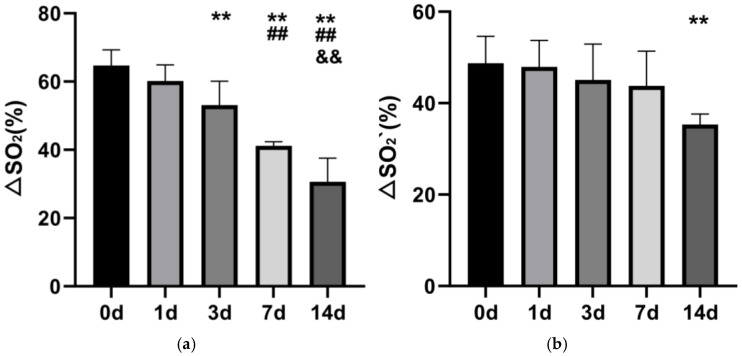
Changes in theoretical oxygen-release capacity of stored rat RBCs (*n* = 6) in the simulated plain environment (**a**) and plateau environment (**b**). **: *p* < 0.01 vs. 0 d; ##: *p* < 0.01 vs. 3 d; &&: *p* < 0.01 vs. 7 d.

The calculated theoretical oxygen-release values of the stored RBCs in the simulated plain environment at 0 d, 1 d, 3 d, 7 d, and 14 d were (64.69 ± 4.62)%, (60.18 ± 4.71)%, (53.10 ± 7.03)%, (41.18 ± 1.19)%, and (30.63 ± 6.95)%, respectively (Figure 9a), exhibiting a declining trend. Theoretical oxygen-release values at 3 d, 7 d, and 14 d were significantly lower than those at 1 d (*p* < 0.01), values at 7 d and 14 d were significantly lower than those at 3 d (*p* < 0.01), and the value at 14 d was significantly lower compared to 7 d (*p* < 0.01). The calculated theoretical oxygen-release values of the stored RBCs in the simulated plateau environment at 0 d, 1 d, 3 d, 7 d, and 14 d were (48.72 ± 5.88)%, (47.94 ± 5.78)%, (45.08 ± 7.83)%, (43.77 ± 7.58)%, and (35.32 ± 2.31)%, respectively (Figure 9b). The values between 0 d and 7 d did not differ significantly (*p* > 0.05), while the value at 14 d was significantly lower than at 0 d (*p* < 0.01).

## 4. Discussion

Oxygen transport is a basic function of blood in the circulatory system. The oxygen obtained from the lungs is transported by blood to various organs and cells, with 98.5% of the oxygen carried in the form of oxygenated hemoglobin. Consequently, the quantity and quality of RBCs (hemoglobin) are crucial for maintaining an adequate oxygen supply to the body. Although the quantity of stored RBCs can be readily determined by counting, there is still a lack of standardized criteria for assessing the efficiency of oxygen supply, a parameter that reflects RBC quantity. It is widely accepted that the in vivo oxygen transport of stored RBCs is modulated by critical factors including 2,3-DPG depletion, RBC deformability, microvascular flow dynamics, and tissue transit time. Certain researchers have used P_50_ values, which indicate oxygen affinity, to evaluate the oxygen supply efficiency of RBCs. For example, oxygen consumption after transfusion can be calculated based on the P_50_ changes in stored and rejuvenated RBCs. Another study categorized stored RBCs into senescent and young groups and found that senescent RBCs exhibited lower P_50_ than young RBCs 14 days after storage, contributing to poor oxygen utilization in tissues after transfusion of senescent RBCs. In the studies mentioned, P_50_ was directly utilized as a marker for the oxygen supply efficiency of stored RBCs [25]. However, the oxygen supply efficiency of blood can also be influenced by other factors such as cooperativity and the Bohr effect in hemoglobin, or even ambient oxygen partial pressure (PO_2_). Hence, it is necessary to analyze these influencing factors, develop a parameter set that can define oxygen supply efficiency, and clarify the roles of these various factors. To include these influencing factors in the evaluation of RBCs’ quality, this study aimed to establish a parameter system for characterizing the oxygen supply efficiency of stored RBCs based on parameters like P_50_, Hill coefficient, SI, and theoretical oxygen-release capacity.

P_50_ values in the present study were obtained through automatic plotting of the ODCs [26]. Our results indicated that the P_50_ values of Tibetans were lower than those of Han Chinese, which was consistent with the findings of previous studies [27]. Cooperativity is an important physiological regulatory mechanism in the oxygen binding/release process of hemoglobin and represents the positive/negative regulatory effects of the various ligands of hemoglobin on oxygen binding. The strength of such regulatory effects can be characterized by the Hill coefficient, which is determined by fitting data points on the ODC to the Hill equation [28]. In previous research, ODCs were usually plotted based on a number of known measurement points, thereby resulting in a limited number of data points that can be used for fitting and a poor goodness of fit. By contrast, the automatic plotting of ODCs in the present study led to the generation of thousands of data points, which enhanced the accuracy of data fitting to a certain extent. Considering that the initial and terminal sections of the S-shaped ODC of hemoglobin differed considerably from the middle section and were unsuitable for fitting to the Hill equation, the present study optimized the fitting range and compared the fitting results of different data ranges. Ultimately, the SO_2_ range of 20–80% was selected for data fitting to the Hill equation, and the exclusion of influences of the initial and terminal curve sections led to goodness of fit values that exceeded 0.990. The calculated Hill coefficient for Han Chinese blood in this study was 2.27 ± 0.21, which was in good agreement with the theoretical prediction [29]. In other words, Tibetan blood possesses higher oxygen affinity and cooperativity than Han Chinese blood, which aids in the binding of oxygen from the external environment to hemoglobin for the maintenance of oxygen supply to the body [30,31]. Both the P_50_ and Hill coefficient values of Dex20-bHb were lower than those of bHb. This can be ascribed to the greater stability of the tetrameric structure of hemoglobin following crosslinking with dextran, which reduced the ease of occurrence of conformational changes and lowered the cooperativity of hemoglobin. These results demonstrate that the Hill coefficient measurement method developed in this study was capable of accurately reflecting changes in the cooperativity of the samples. In addition, Dex20-bHb exhibits minimal sensitivity to chloride ions (Cl^−^) and is not regulated by 2,3-diphosphoglycerate (2,3-DPG) [18]. Incorporating this unique agent into the model significantly broadens the scope of validation. The contrasting characteristics and differential regulatory responses of Dex20-bHb, compared to normal erythrocytes, are pivotal in enriching this parameter system. Its inclusion serves to illustrate that, within the calculated parameter system, the parameters exhibit non-linear correlations and comprehensively represent various aspects of oxygen supply efficiency.

The Bohr effect, which refers to the changes in the oxygen affinity of hemoglobin in response to the pH environment, also serves as an important physiological regulatory mechanism in the oxygen binding/release processes of hemoglobin. In the previous literature, the Bohr effect is usually characterized by the Bohr factor, which is the slope of the straight line obtained when linear fitting is performed with pH on the x-axis and log P_50_ on the y-axis (Φ = ΔlogP_50_/ΔpH) [32,33]. Besides being cumbersome, this method is merely capable of providing an average Bohr factor. In fact, the Bohr effect varies among different pH ranges. Guan et al. reported that SI, calculated based on the relative changes in P_50_ in various pH environments, can characterize the Bohr effect effectively [34]. This proposed method offers easier calculations and greater intuitiveness. Considering the normal blood pH range is 7.36–7.44, with an average of 7.40, we selected pH = 7.4 to simulate the standard body pH environment, pH = 7.2 for tissue cell environments, and pH = 7.6 for lung environments. These pH values were chosen based on a study that used pH values of 7.15 and 7.65 for Bohr factor calculations, with minor adjustments. SI was then calculated by determining the P_50_ values in these different pH settings. This method facilitates the calculation of relative changes in any oxygen carrier’s ODC across varying pH ranges, reflecting different external and internal conditions, and serves as a basis for assessing the Bohr effect. Results revealed that the SI of Tibetan blood was significantly higher than that of Han Chinese blood, likely due to natural selection favoring adaptations to hypoxic conditions in plateau. Additionally, the SI of Dex20-bHb was significantly lower than that of bHb, indicating that the modified hemoglobin, changing its natural conformation, was less regulated by the body’s pH and thus decreased the Bohr effect, aligning with the reduced regulation by allosteric factors reported previously [18]. These findings confirm that the SI calculation method effectively characterizes the Bohr effect in hemoglobin.

In this study, we included ambient PO_2_ and changes in the pH environment as influencing factors in the calculation of the theoretical oxygen-release capacity. Our results showed that in the simulated plain environment, the theoretical oxygen capacity was lower in Tibetan blood than in Han Chinese blood, and lower in Dex20-bHb than in bHb. Calculations conducted with the incorporation of plateau environmental conditions indicated that the trend of the values for Dex20-bHb and bHb remained unchanged, but the theoretical oxygen capacity of Tibetan blood was higher than that of Han Chinese. In other words, Tibetan populations exhibited higher tolerance to hypoxia in plateau environments, which aligns well with theory. These results suggest that oxygen supply efficiency is not solely influenced by oxygen affinity, but also by the collective effects of cooperativity, the Bohr effect, and environmental factors.

Relationships among the various parameters were elucidated by comparing Han Chinese blood, Tibetan blood, bHb, and Dex20-bHb samples. Multiple regression analysis further validated the influences of oxygen affinity, cooperativity, the Bohr effect, and environmental factors on hemoglobin’s oxygen supply efficiency. Results indicated that cooperativity was the primary influencing factor. Under standard environmental conditions, namely normal ambient PO_2_, oxygen affinity’s influence exceeded that of the Bohr effect. However, in high-altitude environments, characterized by lower ambient PO_2_, the Bohr effect’s influence became more pronounced. This may be attributed to hypoxia-induced hyperventilation at plateau, which increases blood alkalinity.

The developed parameter set for the characterization of oxygen supply efficiency was subsequently employed for the assessment of stored rat RBCs. In this study, we validated the parameter system using stored rat red blood cells (RBCs). Compared to human blood, this model offers the advantages of more readily available and a shortened overall testing timeline. More importantly, we referenced multiple established study models and synthesized their preparation methods and storage conditions. Furthermore, the existing literature indicates that changes in stored rat RBCs within 14 days parallel those observed in stored human RBCs over 35 days [35,36,37,38].

Our results revealed that with the prolongation of storage time, the oxygen affinity of the stored blood became elevated, while P_50_ decreased rapidly by approximately 50% within 0–7 d but stabilized thereafter, which was generally consistent with previously reported results in the literature [6,39]. Cooperativity (Hill coefficient) and the Bohr effect (SI) showed an initial trend of increase followed by a decrease from 0 d to 3 d and 7 d of storage, respectively; similar findings have not yet been reported in the literature. The calculated theoretical oxygen-release capacity in the plain environment exhibited a gradual decline but was generally higher than that in the plateau environment. Meanwhile, the calculated theoretical oxygen-release capacity in the plateau environment only showed a significant decrease towards the end of the storage period. These results suggest that systematic assessment of the oxygen supply efficiency of stored RBCs can enhance the understanding of the quality of stored RBCs, thereby aiding in the formulation of appropriate strategies for the transfusion of stored RBCs. For example, our findings indicated that stored rat RBCs maintained the highest oxygen supply efficiency during the first 0–3 days (14 days for human RBCs) of storage in a plain environment, underscoring the need to transfuse RBCs with a short storage duration for critically ill patients experiencing significant blood loss. When transfusing RBCs stored for longer periods, it is important to consider not only the increase in hemoglobin concentration in the patient but also the possible need for increased transfusion volume to achieve the desired oxygen supply effect. In this study, the oxygen affinity, cooperativity, and Bohr effect of hemoglobin were integrated into a parameter system for assessing the oxygen supply efficiency of RBCs. The influence of ambient PO_2_ was also considered, enhancing the accuracy of assessments, which supports the determination of stored RBCs’ quality and prediction of transfusion outcomes.

## 5. Conclusions

This oxygen supply efficiency assessment is a proof-of-concept tool for monitoring the quality of stored RBCs in clinical settings. It can help optimize the quality evaluation system for stored RBCs and will also be applicable to the quality evaluation of hemoglobin products, such as HBOCs, in the future.

## 6. Limitations and Future Study Directions

### 6.1. RBC Morphology and Biomechanics Are the Influencing Factors of Oxygen Transport

The morphology and biomechanical properties of red blood cells (RBCs) significantly influence transfusion efficacy by determining their ability to perfuse the microcirculation, maintain oxygen delivery capacity, and survive in the recipient’s circulation. Abnormal RBC morphologies directly impair transfusion effectiveness. Spherocytes exhibit severely diminished deformability, leading to microvascular occlusion and hemolysis. RBC biomechanics, particularly deformability and membrane elasticity, are critical determinants of transfusion success. Prolonged storage of RBCs (beyond 14 days) induces progressive morphological and biomechanical deterioration and 2,3-DPG loss (reduces oxygen release capacity), impairing oxygen delivery due to left-shifted oxygen dissociation curves [40,41,42]. In the present study, we characterized the oxygen supply efficiency of stored RBCs via theoretical calculation from the perspective of hemoglobin structure and function in vitro, without considering the influence of RBC morphology and biomechanics. To accurately evaluate oxygen supply efficiency in vivo, factors such as deformability and morphology must be taken into account simultaneously.

### 6.2. Human RBCs Should Be Used to Study the Oxygen Supply Efficiency During Storage

In this study, we validated the parameter system using stored rat RBCs. Compared to human blood, this model offers the advantages of greater availability and a shortened overall testing timeline. Although the stored rat RBCs have been proved exhibiting comparable features with the stored human RBCs [35,37,38,43,44], a comprehensive evaluation using human blood is essential. To remedy the inadequacies, we are currently conducting a study to verify the oxygen supply efficiency of the stored human RBCs. The results will be published in the future to address the limitations of the present work.

### 6.3. The Simulated Oxygen Supply Efficiency Assessment Is a Relative Functional Index

While P_50_ is a well-established indicator of blood oxygen delivery, other factors—including the Hill coefficient, the Bohr effect, and environmental conditions—also play key roles. Therefore, the parameter system established in this study provides a systematic characterization of these factors. This approach offers a more detailed and comprehensive evaluation method for the oxygen supply efficiency of stored RBCs, serving as a crucial complement to existing single-parameter characterization. For instance, via the present oxygen supply efficiency, the Hill coefficient, Bohr effect of samples, and their weight in the theoretical oxygen-release capacity could be obtained concurrently. And it clearly identifies which parameters are more critical for maintaining oxygen release-delivery capacity in different environments. However, the method is rudimentary and the data must be compared with the reference standard to illustrate the trend of change, especially the theoretical oxygen-release capacity. Therefore, the present oxygen supply efficiency assessment is a relative functional index, rather than a fully validated predictor of storage RBCs, which need more samples and data to promote the comprehensiveness and accuracy.

## Data Availability

The original contributions presented in this study are included in the article/Supplementary Materials. Further inquiries can be directed to the corresponding authors.

## Supplementary Materials

The following supporting information can be downloaded at https://www.mdpi.com/article/10.3390/life16020205/s1, Table S1. Anova table for regression Equation (7); Table S2. Anova table for regression Equation (8); Table S3. Coefficients for regression Equation (7); Table S4. Coefficients for regression Equation (8).
