# Supplementary material for: Simulated Oxygen Supply Efficiency Assessment to Represent Stored Red Blood Cells Quality
Source: Life (Basel). 2026 Jan 26;16(2):205. doi: 10.3390/life16020205 (PMC12941439; doi:10.3390/life16020205)
Supplement: Supplementary file 1 [file life-16-00205-s001.zip › life-4060861-supplementary.pdf]

Supporting information

Simulated Oxygen Supply Efficiency Assessment to Represent Stored Red Blood Cells Quality

Authors: Zongtang Chu, Guoxing You, Weidan Li, Peilin Shu, Dong Qin,
Lian Zhao *, Hong Zhou, Ying Wang *

Author affiliations: Academy of Military Medical Sciences, Beijing 100850, China

Correspondence: zhaolian@bmi.ac.cn (L.Z.); wy830111@163.com (Y.W.)

Table S1. Anova table for regression equation (7)

source	*SS*	*df*	*MS*	*F*	p-value
Regression	209.704	3	69.901	145.253	0.001***
Error	4.812	9	0.481		
Total	214.516	12			

Response variable: ΔSO_2, X_1: P_{50}; X_2: Hill coefficient; X_3: SI

Predictor variable: P_{50}, Hill coefficient, and SI

SS=Sum of Squares, *df*=degrees of freedom, *MS*= Mean Square, *F*=F-statistic

Table S2. Anova table for regression equation (8)

source	*SS*	*df*	*MS*	*F*	p-value
Regression	254.914	3	84.971	143.63	0.01**
Error	5.324	9	0.592		
Total	260.238	12			

Response variable: $\Delta SO_2'$, X_1: P_{50}; X_2: Hill coefficient; X_3: SI

Predictor variable: P_{50}, Hill coefficient, and SI

SS=Sum of Squares, *df*=degrees of freedom, *MS*= Mean Square, *F*=F-statistic

Table S3. coefficients for regression equation (7)

	B	*SE*	*β*	*t*	p
Intercept	5.501	1.276	-	4.313	0.01**
X_1	0.588	0.017	0.263	2.382	0.01**
X_2	10.839	2.323	0.798	4.666	0.01**
X_3	0.032	0.008	0.067	-3.401	0.01**

Response variable: ΔSO_2

B=Unstandardized Coefficient, *SE*=Standard Error, *β*=Beta Coefficient

Table S4. coefficients for regression equation (8)

	B	*SE*	*β*	*t*	p
Intercept	4.493	1.438	-	3.125	0.01**
X1	0.146	0.001	0.119	3.693	0.01**
X2	2.152	0.354	0.133	2.494	0.01**
X3	0.511	0.148	0.746	3.444	0.01**

Response variable: $\Delta SO_2'$

B=Unstandardized Coefficient, *SE*=Standard Error, *β*=Beta Coefficient
